# MicroRNA-17-92 Regulates the Transcription Factor E2F3b during Myogenesis In Vitro and In Vivo

**DOI:** 10.3390/ijms18040727

**Published:** 2017-03-31

**Authors:** Zhixiong Tang, Nian Liu, Lan Luo, Kang Kang, Li Li, Ruiyang Ni, Huiling Qiu, Deming Gou

**Affiliations:** 1 Shenzhen Key Laboratory of Microbial Genetic Engineering, College of Life Sciences, Shenzhen University, Shenzhen 518060, China; zhizhi0719s@163.com (Z.T.); liunian27@hotmail.com (N.L.); lanluochen@hotmail.com (L.L.); ylili@szu.edu.cn (L.L.); 2 Department of Biochemistry and Molecular Biology, School of Basic Medical Sciences, Shenzhen University Health Sciences Center, Shenzhen 518000, China; kangkang@szu.edu.cn; 3 School of Life Sciences, Peking University, Beijing 100871, China; niruiyang@pku.edu.cn; 4 Biomedical Engineering, College of Health and Environmental Engineering, Shenzhen Technology University, Shenzhen 51000, China

**Keywords:** miR-20a, E2F3b, muscle

## Abstract

Myogenic differentiation, which occurs during muscle development, is a highly ordered process that can be regulated by E2F transcription factors. Available data show that E2F3b, but not E2F3a, is upregulated and required for myogenic differentiation. However, the regulation of E2F3b expression in myogenic differentiation is not well understood. To investigate whether E2Fb expression is controlled by miRNAs, we used bioinformatics to combine the database of microRNAs downregulated during myogenesis and those predicted to target E2F3. This identified miR-17 and miR-20a as miRNAs potentially involved in E2F3 regulation. We found that miR-17-92 controls the expression of E2F3b in C2C12 cells during myogenic differentiation. Moreover, we confirmed that miR-20a regulates the expression of E2F3b proteins in vivo using a muscle regeneration model.

## 1. Introduction

Skeletal muscle differentiation occurs during muscle growth and regeneration [1,2]. This process can be experimentally induced by forcing the myoblasts to undergo differentiation with 2% horse serum, which causes exit of the cell cycle, then allowing myocyte fusion into myotubes [3]. It is well known that myogenesis is highly regulated by myogenic regulatory factors including myogenic factor 5 (Myf5), MyoD, myogenin, and myogenic regulatory factor 4 (MRF4). E2F3 knockout causing a defect in heart muscle development suggests that the E2F3 transcription factor is involved in muscle differentiation [4]. The mouse E2F3 locus expresses two isoforms, E2F3a and E2F3b. These isoforms differ in their first exon, which encodes 122 and six amino acids, respectively; the remaining coding sequences are identical [5,6]. These two isoforms have functional complementation, and it is difficult to investigate their respective functions [7]. However, the expression of the two isoforms differ, with E2F3a highly expressed in proliferative cells, especially those in the G1 phase to S phase transition, while E2F3b is expressed during the whole cell cycle and in G0 cells [8]. Available data shows that E2F3a and E2F3b have opposing roles in cell cycle and differentiation. Asp et al. previously used a chromatin immunoprecipitation (ChIP)-on-ChIP assay and acute ablation analysis in C2C12 cells and found that the genes regulated by E2F3a and E2F3b are associated with proliferation and myogenic differentiation, respectively [9]. However, the regulation of E2F3b expression in myogenic differentiation is not well understood.

MicroRNAs (miRNAs) are 21~23-nucleotide, noncoding RNA molecules involved in post-transcriptional regulation through targeting mRNAs to inhibit its expression [10]. In the present study, we analysed the expression patterns of 720 miRNAs in mouse myoblasts and differentiated myotubes to screen for the miRNAs that potentially regulate E2F3b protein expression during myogenesis. Target gene prediction suggested that miR-17-92clusters, including miR-17 and miR-20a, regulate E2F3b. In the present study, we identified E2F3b as a common target of miR-17 and miR-20a and revealed how miR-17-92 regulates E2F3b during myogenesis in vitro and in vivo.

## 2. Results

### 2.1. E2F3b Is Upregulated during Myogenesis and Is Essential for Myogenic Differentiation

**Figure 1:**
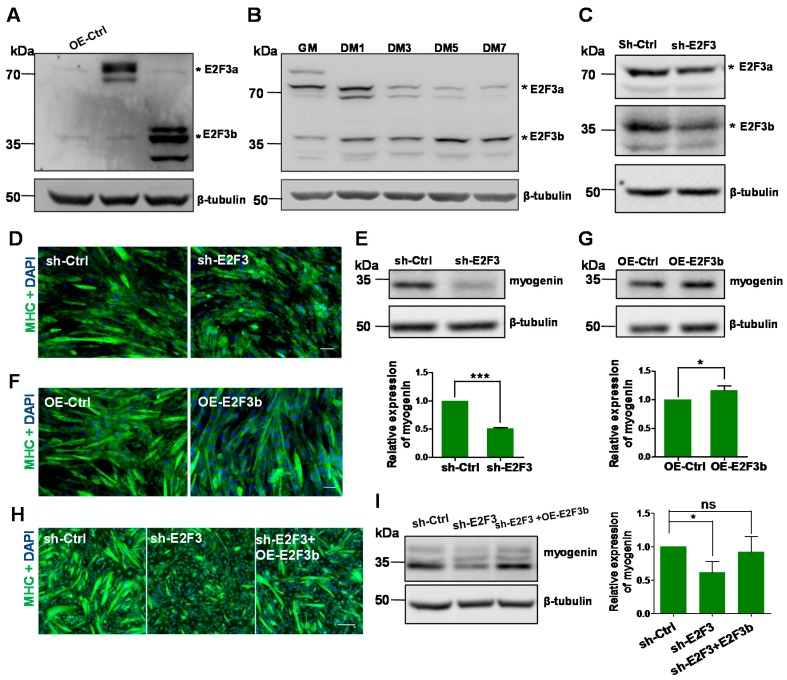
E2F3b is upregulated during myogenesis and is essential for myogenic differentiation.(**A**) Western blotting showing increased expression of E2F3a and E2F3b protein in C2C12 myoblasts transfected with vectors expressing E2F3a or E2F3b (OE-E2F3a, OE-E2F3b), compared with the control (empty vector, OE-Ctrl). β-tubulin was used as a loading control; (**B**) Western blotting showing the expression of E2F3a and E2F3b during myogenic differentiation. C2C12 cells were cultured in growth medium (GM) and switched to differentiation medium (DM) for one to seven days. β-tubulin was used as a loading control; (**C**) Western blotting confirmed the efficiency of sh-E2F3 on E2F3a and E2F3b protein expression in C2C12 cells. β-tubulin was used as a loading control; (**D**) Immunostaining of myosin showing that silenced E2F3b resulted in smaller myotubes. C2C12 cells were infected with sh-E2F3 vectors in DM for three days. Scale bar, 200 μm; (**E**) Western blotting showing that silenced E2F3b resulted in a decreased myogenin level at DM3. β-tubulin was used as a loading control. Protein quantitation of myogenin was performed with Image J software. The error bars depict the mean ± SD of three cell samples; (**F**) Immunostaining of myosin showing that OE-E2F3b resulted in longer myotubes. C2C12 cells were lentivirally infected with vectors expressing OE-E2F3 in DM for three days. Scale bar, 200 μm; (**G**) Western blotting showing that OE-E2F3b resulted in increased myogenin protein at DM3. β-tubulin was used as a loading control. Protein quantitation of myogenin was performed with Image J software. The error bars depict the mean ± SD of three cell samples; (**H**) Lentiviral E2F3b rescues the formation of myotubes in sh-E2F3 cells. sh-E2F3 cells were lentivirally infected by E2F3b, transferred to DM, and stained for Myosin heavy chain (MHC) and 4′,6-diamidino-2-phenylindole (DAPI) at DM4; (**I**) Western blotting shows that the introduction of E2F3b increases myogenin protein expression at DM4. Scale bar, 500 μm. The error bars depict the means ± SD of three independent samples. * *p* < 0.05, *** *p* < 0.001 and ns, non-significant.

To validate the antibody for the detection of E2F3a and E2F3b, we transducted pLVX-E2F3a and pLVX-E2F3b, respectively, into C2C12 cells and measured their protein levels by western blotting. As shown in Figure 1A, both E2F3a and E2F3b can be well recognised by the same antibody. Then, we determined the protein levels of E2F3a and E2F3b in C2C12 cells during myogenesis. As shown in Figure 1B, E2F3a was predominantly expressed in growth medium (GM), then gradually reduced. After the cells had been induced to differentiate by differentiation medium (DM), E2F3a expression was maintained at a very low level or was undetectable. In contrast, E2F3b expression gradually increased from GM to DM7. These data indicated that the upregulation of E2F3b is associated with myogenic differentiation.

To confirm a role for E2F3 in myoblast differentiation, we constructed the sh-E2F3 lentiviral vector and verified its efficiency by real-time PCR (Figure S1) and western blotting (Figure 1C). Consistent with a previous study of Asp et al. [9], the formation of myotube was severely damaged when E2F3 was silenced (Figure 1D). The effect of E2F3 silencing on myogenic differentiation was further confirmed by western blotting, as indicated by a decrease of myogenin protein in sh-E2F3 C2C12 cells at DM3 (Figure 1E). These results suggested that E2F3 plays a critical role in myogenic differentiation. We further lentivirally infected the C2C12 with pLVX-E2F3b and subsequently transferred the myoblasts to DM for three days. Immunofluorescence results showed that myotube fusion levels were slightly higher in the E2F3b overexpressed group when compared with the control (OE-Ctrl) (Figure 1F). Its pro-differentiation effect was confirmed by western blotting for myogenin (Figure 1G). We next confirmed the positive function of E2F3b in myogenic differentiation by infecting sh-E2F3 C2C12 cells with a lentivirus overexpressing E2F3b. Immunostainings for myosin showed that E2F3b rescued the formation of myotube in sh-E2F3b cells (Figure 1H). Moreover, we found that the lentiviral introduction of E2F3b resulted in an increase of myogenin protein in sh-E2F3 cells (Figure 1I). Taken together, these results suggest that the upregulation of E2F3b during myoblast differentiation is essential for the positive regulation of myogenesis.

### 2.2. E2F3b Expression Is Regulated by miR-17 and miR-20a during Myogenesis In Vitro

**Figure 2:**
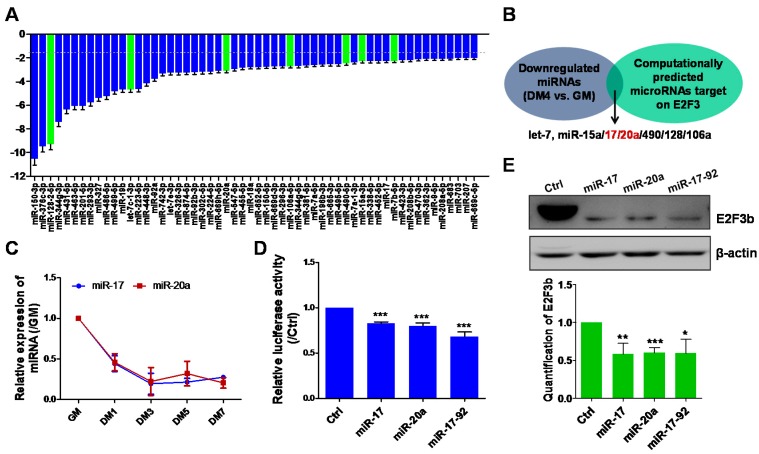
E2F3b expression is regulated by miR-17-92 in vitro. (**A**) Quantitative real-time PCR (RT-PCR) showing that 56 miRNAs were downregulated in differentiated C2C12myotubes at DM4, compared with myoblasts at GM. The miRNAs computationally predicted to target E2F3b were shown in green; (**B**) Seven miRNAs potentially regulated the expression of E2F3b, including let-7c, miR-15a, miR-17, miR-20a, miR-106a, miR-128, and miR-490; miR-17 and miR-20a, both members of the miR-17-92 cluster were shown in red. (**C**) Quantitative RT-PCR was performed to analyse the expression of miR-17 and miR-20a in C2C12 cells during myogenic differentiation. The data are presented as the means ± SD (*n* = 3), the expression in GM was set to 1.0; (**D**) 3′UTR reporter assay showing that miR17/20a and miR-17-92 targeted the 3′UTR of E2F3 in HEK293A cells. The data are presented as the means ± SD (*n* = 3). *** *p* < 0.001; (**E**) miR17/20a and miR-17-92 downregulated the protein expression of E2F3b in differentiated C2C12 cells at DM4. β-actin was used as a loading control. Protein quantitation of E2F3b was performed with Image J software. Values are means ± SD. Data are representative of three independent cell samples. * *p* < 0.05, ** *p* < 0.01, *** *p* < 0.001.

Increasing evidence suggests that microRNAs (miRNAs) play a critical role in regulating myogenesis. Because E2F3b is upregulated during C2C12 cell myogenesis, miRNAs directly targeting E2F3b would be expected to be downregulated. We therefore analysed the expression profiles of 720 miRNAs in myogenesis. Figure 2A shows all downregulated miRNAs, with those predicted to target E2F3b by bioinformatics analysis represented in green (Table S1).The expression of E2F3b was potentially regulated by seven miRNAs including let-7c, miR-15a, miR-17, miR-20a, miR-106a, miR-128, and miR-490 (Figure 2B). Among them, miR-17 and miR-20a from the miR-17–92 cluster were significantly downregulated and further confirmed by time-dependent assay (Figure 2C). Therefore, there was a reverse relationship between the expression of miR-17/20a and E2F3b during myogenesis. Then, we performed 3′UTR luciferase assay in C2C12 cells by co-transfecting E2F3b 3′UTR reporter vector and miR-17, miR-20a, or miR-17-92 overexpression vector. Compared to the control vector, E2F3 3′UTR luciferase activity was significantly reduced by miR-17, miR-20a, or miR-17-92 (Figure 2D). Moreover, overexpression of miR-17, miR-20a, or miR-17-92 in differentiated C2C12 cells (DM4) dramatically reduced the expression of E2F3b protein (Figure 2E). Collectively, these results showed that E2F3b is a direct target of miR-17/20a in C2C12 cells.

### 2.3. miR-20a Regulates E2F3b Expression In Vivo

**Figure 3:**
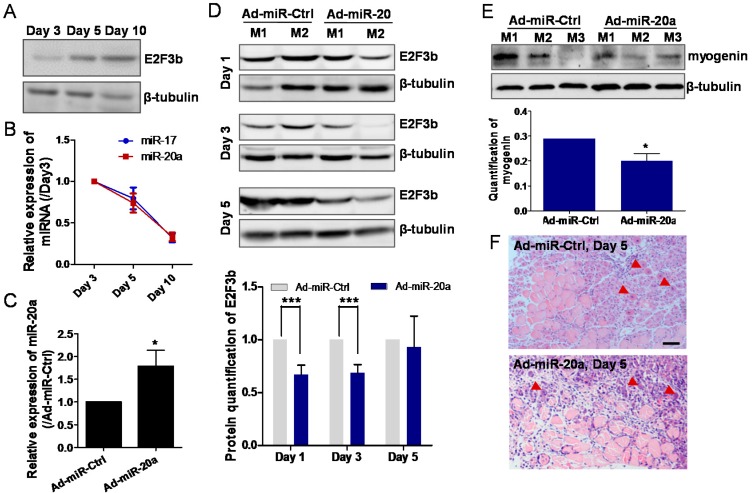
miR-20a regulates E2F3b expression in vivo. (**A**) Western blotting showing that E2F3b protein was upregulated during muscle regeneration after cardiotoxin (CTX)-injury; (**B**) Quantitative RT-PCR was performed to analyse the expression of miR-17 and miR-20a on 3, 5, and 10 days post CTX-injury. The data are presented as the means ± SD. Data are representative of three independent samples; (**C**) Relative expression of miR-20a in Ad-miR-20a mice compared with Ad-miR-NC mice one day before CTX-injury. The data are presented as the means ± SD (*n* = 6); (**D**) Western blotting for E2F3b of mice injected with Ad-miR-Ctrl or Ad-miR-20a 1, 3, and 5 days post CTX-injury. M1, mouse 1; M2, mouse 2. β-tubulin was used as a loading control. Quantification of E2F3b protein levels was from five mice; (**E**) Western blotting showing Ad-miR-20a injection reduced the protein expression of myogenin in tibialis anterior muscles on day 5 after the CTX-injury. Quantification of myogenin protein was from five mice. The data are presented as the means ± SD. * *p* < 0.05 and *** *p* < 0.001. (**F**) Hematoxylin and eosin (H & E) images of the tibialis anterior muscle cross sections of mice on day 5 post-CTX injury, showing a comparison between injection of Ad-miR-20a or control (Ad-miR-NC). Arrows indicate the regenerating myofibers. Scale bar, 200 μm.

To investigate E2F3b and miR-20a in vivo, we constructed a muscle regeneration model by injecting cardiotoxin (CTX) into the tibialis anterior muscles of mice to mimic myogenesis in vivo [11]. Hematoxylin and eosin (H & E) staining showed that muscle fibers were seriously damaged by CTX treatment. Following the injury, the muscle satellite cells were activated and differentiated to form new myofibers to repair damaged myofibers (Figure S2). We found that E2F3b protein level was increased (Figure 3A), while miR-17 and miR-20a were downregulated during muscle regeneration (Figure 3B). This is consistent with the results in vitrothat miR-17 and miR-20a are negatively correlated with the expression of E2F3b protein.

We further injected adenovirus vectors expressing miR-20a (Ad-miR-20a) or control (Ad-miR-Ctrl) into the tibialis anterior muscles of mice 10 days before CTX-injury. Figure 3C showed the successful overexpression of miR-20a in mouse tibialis anterior muscles. Compared to Ad-miR-Ctrl, E2F3b was significantly downregulated on day 1 and 3 with less obvious change on day 5 post CTX-injury (Figure 3D). Western blotting to assess the effect of miR-20a on muscle regeneration showed that myogenin was downregulated compared with controls (Figure 3E). Moreover, analysis of cross-sectional areas revealed that new myofibers containing central nuclei were smaller in Ad-miR-20a-injected mice compared with the controls (Figure 3F). Therefore, Ad-miR-20a repressed the muscle differentiation and delayed the growth of new myofibers. Taken together, these results show that the levels of miR-20a and E2F3b are negatively correlated during mouse muscle regeneration, and that miR-20a overexpression inhibits E2F3b expression leading to impaired muscle regeneration in vivo.

## 3. Discussion

Our study shows that E2F3b is indispensable in myogenic differentiation, and suggests for the first time that E2F3b is regulated by miR-17 and miR-20a during C2C12 cell myogenesis. More importantly, we report that E2F3b and miR-20a levels are negatively correlated in a muscle regeneration model and that miR-20a overexpression inhibits the expression of E2F3b in vivo and delays muscle differentiation.

We revealed that miR-17-92 regulates E2F3b during myogenesis, and thus promotes muscle differentiation in vivo. However, other miRNAs can also target E2F3. Our screening of miRNAs that target E2F3 and are downregulated in myogenic differentiation also identified miR-15a, miR-490, miR-128, miR-106a, and let-7. Previous studies have shown that miR-128 targets E2F3 and inhibits cell proliferation in glioma [12]. The remaining miRNAs have not been investigated in our study, so they have potential research value as novel miRNAs that may regulate E2F3 in myogenesis. On the other hand, miR-20a regulates muscle development by targeting multiple genes. It has been previously showed that the miR-17-92 cluster targets ENH1 to modulate myoblast proliferation and differentiation [11]. Additionally, Sjogren et al. identified 32 genes that are potentially targeted by miR-20a during human skeletal differentiation in muscle cell [13]. Further in-depth studies of miR-20a regulatory mechanisms involving these targets should be conducted.

In this study, we were unable to construct separate shRNA vectors for E2F3a and E2F3b because of the limited difference in base sequences between E2F3a and E2F3b, although some researchers have previously used E2F3a- and E2F3b-specific knockouts to explore their functions [7]. In our study, silencing of E2F3 by sh-E2F3 inhibited both E2F3a and E2F3b expression, as shown by western blotting assays. Because expression of E2F3a was strongly downregulated in differentiating muscle cells, the blockage of myotube formation and downregulation of myogenin expression following E2F3 silencing was caused at least partly by the reduced expression of E2F3b.

E2F3 reversibly regulates the expression of miR-17-92. The existence of an autoregulatory feedback loop between E2F factors and miRNAs from the miR-17-92 cluster has been proposed [14]. Woods et al. previously analysed the promoter structure of the miR-17-92 family and showed that E2F3 was the primary E2F family member that occupies the promoter [15]. Later studies proved that E2F1 and E2F3 activate the transcription of miR-17-92 [15,16]. More recently, E2F1 has been proposed as a positive regulator of miR-17-92 during muscle differentiation. In our previous study, we found that E2F1 was also downregulated during muscle regeneration and exhibited a significant positive correlation of expression with miR-17 and miR-20a [11]. However, it remains to be determined whether E2F3b is involved in the transcriptional regulation of miR-17-92 in myoblast differentiation.

## 4. Materials and Methods

### 4.1. Cell Culture

C2C12, HEK293A, and HEK293T cells were purchased from the American Type Culture Collection (ATCC, Manassas, VA, USA). C2C12 cells were maintained in Dulbecco’s modified Eagle medium (DMEM; Corning Cellgro, Manassas, VA, USA) supplemented with 10% fetal bovine serum (FBS; Biowest, Nuaillè, France) until they reached 80–90% confluence. At this point, myogenic differentiation was induced by changing the culture medium to DMEM with 2% horse serum (Gibco, Grand Island, NY, USA) in a humidified incubator with 5% CO_2_ at 37 °C. HEK293A and 293T cells were cultured in DMEM with 10% FBS.

### 4.2. Mouse Muscle Regeneration Model and Adenovirus Injection

All the protocols were approved by the Animal Ethical and Welfare Committee of Shenzhen University (Approval No. AEWC-2014-001004). The male C57BL/6 mice (Guangdong Medical Laboratory Animal Center) were used to generate a mouse regeneration model by injecting cardiotoxin (CTX), as described previously [11]. Adenoviruses expressing miR-20a or a negative control were injected into the tibialis anterior muscles 10 days prior to the CTX injection. Tibialis anterior muscles were collected at day 0, 1, 3, 5, and 10 post-CTX injury for RNA and protein extraction.

### 4.3. RNA Extraction and Quantitative RT-PCR

Total RNA was extracted from cultured C2C12 cells or tibialis anterior muscles using RNAiso Reagent (TaKaRa, Dalian, China) according to the manufacturer’s instructions, and quantified using the NanoDrop2000c Spectrophotometer (Thermo Fisher Scientific, Wilmington, DE, USA). miRNAqRT-PCR was performed using the S-Poly(T) Plus method with SNORNA234 as the mouse miRNA control (Geneups, Shenzhen, China) [17,18]. SYBR Green was used as a quantitative method with RPL-14 as a normalization control. Primers for the detection of miR-17, miR-20a, and E2F3b are listed in Table S2.

### 4.4. Plasmids

#### 4.4.1. E2F3b Overexpression and Inhibition

To construct overexpression plasmids, the open reading frame of mouse E2F3a was synthesized by Viewsolid Biotech (Beijing, China) and E2F3b was amplified from C2C12 myoblast cDNA using the primer listed in Table S3. The sequences were further cloned into the pLVX-puro plasmid (Clontech) through *EcoR*I/*Xho*I restriction sites. Empty vector was used as overexpression control. Plasmid vectors containing shRNA targeting E2F3 (sh-E2F3) or a nonspecific control (sh-Ctrl) were constructed based on pLVX-hU6. shRNA sequences containing *Bsm*BI restriction site were synthesized using primers listed in Table S3.

#### 4.4.2. 3′UTRLuciferase Reporter Assay

To construct the E2F3 3′UTR luciferase reporter plasmid, the E2F3 3′UTR was amplified from mouse genomic DNA using the primers listed in Table S3. The purified PCR products were then cloned downstream of the luciferase reporter gene between *Eco*RI/*Xho*I restriction sites of the miRGlo vector (Promega, Madison, WI, USA).

#### 4.4.3. miRNAOverexpression

Lentiviral vectors overexpressing miR-17, miR-20a, and miR-17-92 and adenoviral vectors (Ad-miR-20a and Ad-miR-NC) were constructed as described previously [11].

### 4.5. Transfection and Luciferase Reporter Assays

The transfection of plasmids into C2C12 cells and E2F3 3′UTR luciferase assays were performed as previously described [11]. Plasmids expressed miR-17, miR-20a, or miR-17-92 and E2F3 3′UTR reporter plasmids were co-transfected into 293A cells.

### 4.6. Immunoblotting, Cell Immunostaining, and Immunohistochemistry

#### 4.6.1. Western Blotting

Total protein isolation was performed as previously described [11]. We collected proteins from C2C12 cells and mouse tibialis anterior muscles. The following primary antibodies were used: β-actin (1:10,000; Proteintech 66009-1-IG, Wuhan, China), β-tubulin (1:5000; Proteintech 10094-1-AP, Wuhan, China), E2F3 (1:600; Santa Cruz Biotechnology SC-878, Santa Cruz, CA, USA), and myogenin (1:5000; Abcamab124800, Cambridge, MA, USA). Horseradish peroxidase-conjugated secondary antibodies (1:10,000; Bio-Rad, Hercules, CA, USA) were also used.

#### 4.6.2. Immunostaining

Cellular immunostaining was carried out using previously reported protocols with slight modifications [19]. Briefly, C2C12 cells were grown in 24-well plates, fixed with 4% formaldehyde in phosphate-buffered saline (PBS) for 20 min and permeabilized with 0.5% Triton X-100 in PBS for 20 min, both at room temperature. Cells were blocked and incubated with primary antibodies for 3 h at room temperature or overnight at 4 °C at the following dilutions: anti-myosin heavy chain (1:100; Abcam) with 1% bovine serum albumin and 0.05% Triton X-100 in PBS. Indirect immunofluorescence was detected after incubation with fluorescein isothiocyanate-conjugated anti-mouse IgG (1:500; Abcam) for 1 h at room temperature. Cell nuclei were stained with 4′,6-diamidino-2-phenylindoledihydrochloride for 5–10 min. After several washes with PBS, the cells were examined under a fluorescence microscope (Zeiss, Oberkochen, Germany).

### 4.7. Statistical Analysis

All results are expressed as the mean of at least three triplicates for each treatment. Pairwise comparisons were performed using a two-tailed Student’s *t*-test with STATGRAPHICS (Centurion XVI.I) software (StatPoint Technologies, Warrenton, VA, USA). A *p*-value of <0.05 was considered statistically significant.

## 5. Conclusions

In conclusion, this study focused on the association between E2F3b and myogenic differentiation, and showed that the miR-17-92/E2f3b axis has an important regulatory role in C2C12 myogenesis and the muscle regeneration model. Our results contribute to the understanding of E2F3b function in myogenesis, and the fundamental mechanism of expression regulation by miR-17-92 in myogenic differentiation.

## Supplementary Materials

Supplementary materials can be found at www.mdpi.com/1422-0067/18/4/727/s1.
