# Supplementary material for: MicroRNA-17-92 Regulates the Transcription Factor E2F3b during Myogenesis In Vitro and In Vivo
Source: Int J Mol Sci. 2017 Mar 31;18(4):727. doi: 10.3390/ijms18040727 (PMC5412313; doi:10.3390/ijms18040727)
Supplement: Supplementary file 1 [file ijms-18-00727-s001.zip › supplementary figure 1 and 2.pptx]

## Slide 1
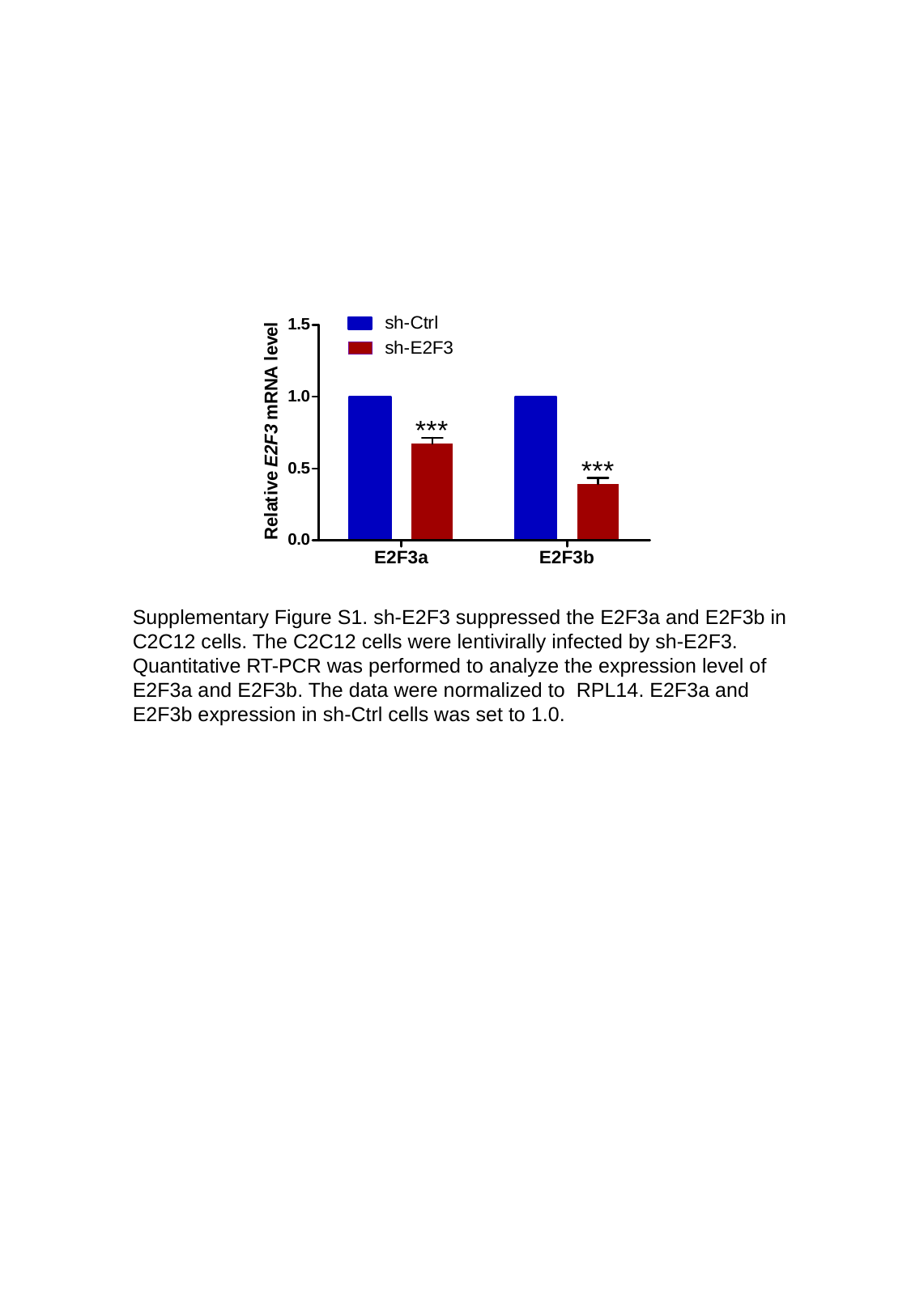

Supplementary Figure S1. sh-E2F3 suppressed the E2F3a and E2F3b in C2C12 cells. The C2C12 cells were lentivirally infected by sh-E2F3. Quantitative RT-PCR was performed to analyze the expression level of E2F3a and E2F3b. The data were normalized to RPL14. E2F3a and E2F3b expression in sh-Ctrl cells was set to 1.0.

## Slide 2
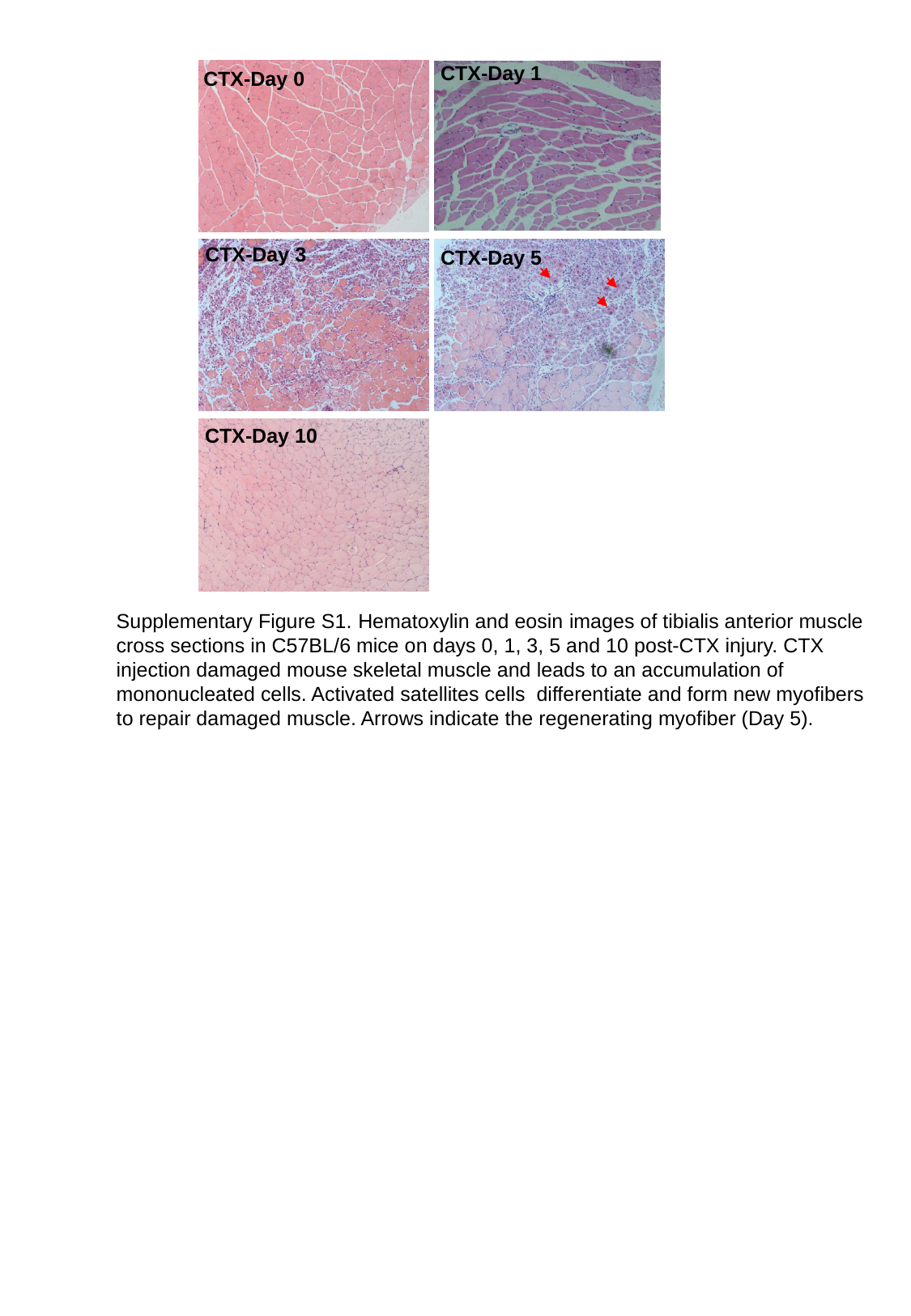

CTX-Day 1
CTX-Day 0
CTX-Day 3
CTX-Day 5
CTX-Day 10
Supplementary Figure S1. Hematoxylin and eosin images of tibialis anterior muscle cross sections in C57BL/6 mice on days 0, 1, 3, 5 and 10 post-CTX injury. CTX injection damaged mouse skeletal muscle and leads to an accumulation of mononucleated cells. Activated satellites cells differentiate and form new myofibers to repair damaged muscle. Arrows indicate the regenerating myofiber (Day 5).
