# Supplementary material for: MicroRNA-17-92 Regulates the Transcription Factor E2F3b during Myogenesis In Vitro and In Vivo
Source: Int J Mol Sci. 2017 Mar 31;18(4):727. doi: 10.3390/ijms18040727 (PMC5412313; doi:10.3390/ijms18040727)
Supplement: Supplementary file 1 [file ijms-18-00727-s001.zip › E2F3-Supplementary Tables.pdf]

Supplementary Table S1.miRNAs predicted to target the 3'UTR of E2F3

miR-34ac/449abc
miR-15abc/16abc/195/322/424/497/1907
miR-10abc
miR-141/200a
miR-490
miR-199ab
miR-25/32/92abc/363/367
miR-200bc/429/548a
miR-125ab/351/670/4319
miR-221/222ab/1928
miR-124ab/506
miR-503
miR-193ab
miR-33ab
miR-148ab/152
miR-30abcdef/384
miR-375
miR-194
miR-128ab
miR-21/590
miR-17/20ab/93/106ab/427/518a/519d
miR-145
miR-142
miR-27abc
miR-31
miR-153
miR-203
let-7/miR-98/4458/4500

Supplementary Table S2. Primers used for quantitative PCR.

mmu-miR-17-FW	5′-TGGCAAAGTGCTTACAGTGC-3′
mmu-miR-17-REV	5′-GTGCAGGGTCCGAGGTCAGAGCCACCTGGGCAATTTTTTTTTTTCTACCT-3′
mmu-miR-20a-FW	5′-TCGGTAAAGTGCTTATAGTGC-3′
mmu-miR-20a-REV	5′-GTGCAGGGTCCGAGGTCAGAGCCACCTGGGCAATTTTTTTTTTTCTACCT-3′
SNORNA234-FW	5'-GATTTAACAAAAATTCGTCACTACCA-3'
SNORNA234-REV	5'-GTGCAGGGTCCGAGGTCAGAGCCACCTGGGCAATTTTTTTTTTTTCTCAG-3'
E2F3b- FW	5'-CCCTTACAGCAGCAGGCAAAGCGAA-3'
E2F3b- REV	5'-GGCTCAGGAGCTGAATGAACTTCTT-3'
RPL14-FW	5'-GGCTTTAGTGGATGGACCCT-3'
RPL14-REV	5'-ATTGATATCCGCCTTCTCCC-3'

Supplementary Table S3.Primers used for plasmids construction.

E2F3-1-shRNA-P1	5'-ACCGCCAAGACCACAATGGGAATATCTCGAGATA-3'
E2F3-1-shRNA-P2	5'-AAAACCAAGACCACAATGGGAATATCTCGAGATA-3'
E2F3-1-shRNA-P3	5'-TTCCCATTGTGGTCTTGG-3'
E2F3-2-shRNA-P1	5'-ACCGGGAAGGCATCCACCTCATTAACTCGAGTTA-3'
E2F3-2-shRNA-P2	5'-AAAAGGAAGGCATCCACCTCATTAACTCGAGTTA-3'
E2F3-2-shRNA-P3	5'-ATGAGGTGGATGCCTTCC-3'
E2F3b-P1	5'-CCACTCGAGATGCCCTTACAGCAGCAGGCAAAGCGAAGGCTGGAGCTAGGAGAAAGCGG-3'
E2F3b-P2	5'-GCTAGGAGAAAGCGGTCATCAGTACCTCTCAGATGGTTTAAAAACCCCCAAGGGCAAAG-3'
E2F3b-P3	5'-CCCAAGGGCAAAGGAAGAGCTGCACTACGAAGTCCAGATAGTCCAAAAAAAAAAACGCG-3'
E2F3b-P4	5'-CGAGGCTCAGGAGATAGTCCTCTTGCAGCAGGGGAGGCAGTAAGTTC-3'
E2F3b-P5	5'-CCAAATCGTAAGCATCGAAGAGATCGCTGATGCCTTCCTCCTCCCCGAGGCTCAGGAGA-3'
E2F3b-P6	5'-GTTGAATTCTCAACTACACATGAAGTCTTCCACCAGTGGGAGCTTTTCCAAATCGTAAG-3'
E2F3-3-UTR- FW	5'-ACTGGAATTCGATCTAAGGTTTATCAGCCTCTGCA-3'
E2F3-3-UTR- REV	5'-GAACCTCGAGTGTACCAAGTCCAGTGTGTGTGAG-3'
